# The Benefits of Social Influence in Optimized Cultural Markets

**DOI:** 10.1371/journal.pone.0121934

**Published:** 2015-04-01

**Authors:** Andrés Abeliuk, Gerardo Berbeglia, Manuel Cebrian, Pascal Van Hentenryck

**Affiliations:** 1 Optimization Research Group, National ICT Australia, Melbourne, Victoria, Australia; 2 Melbourne School of Engineering, The University of Melbourne, Melbourne, Victoria, Australia; 3 Centre for Business Analytics, Melbourne Business School, The University of Melbourne, Melbourne, Victoria, Australia; 4 Research School of Computer Science, The Australian National University, Canberra, ACT, Australia; Cinvestav-Merida, MEXICO

## Abstract

Social influence has been shown to create significant unpredictability in cultural markets, providing one potential explanation why experts routinely fail at predicting commercial success of cultural products. As a result, social influence is often presented in a negative light. Here, we show the benefits of social influence for cultural markets. We present a policy that uses product quality, appeal, position bias and social influence to maximize expected profits in the market. Our computational experiments show that our profit-maximizing policy leverages social influence to produce significant performance benefits for the market, while our theoretical analysis proves that our policy outperforms in expectation any policy not displaying social signals. Our results contrast with earlier work which focused on showing the unpredictability and inequalities created by social influence. Not only do we show for the first time that, under our policy, dynamically showing consumers positive social signals increases the expected profit of the seller in cultural markets. We also show that, in reasonable settings, our profit-maximizing policy does not introduce significant unpredictability and identifies “blockbusters”. Overall, these results shed new light on the nature of social influence and how it can be leveraged for the benefits of the market.

## Introduction

Prediction in cultural markets is a wicked problem: On the one hand, experts routinely fail at predicting commercial success for items such as movies, books, and songs [1–5] while, on the other hand, hit products are generally considered qualitatively superior to the less successful ones [6]. If those hit products are of superior quality, why can’t experts detect them a priori? As Duncan Watts puts it, in cultural markets “everything is obvious … once you know the answer,” as experts find it extremely hard to benefit from their specific knowledge [7].

In their seminal paper [8], Salganik, Dodds, and Watts offer a satisfactory explanation for this paradox: The impact of social influence on the behavior of individuals (word-of-mouth), a process a priori invisible to experts, may distort the quality perceived by the customers, making quality and popularity out of sync and expert predictions less reliable. To investigate this hypothesis experimentally, they created an artificial music market called the MusicLab. Participants in the MusicLab were presented a list of unknown songs from unknown bands, each song being described by its name and band. The participants were divided into two groups exposed to two different experimental conditions: The *independent* condition and the *social influence* condition. In the first group (independent condition), participants were provided with no additional information about the songs. Each participant would decide which song to listen to from a random list. After listening to a song, the participant had the opportunity to download it. In the second group (social influence condition), each participant was provided with an additional information: The number of times the song was downloaded by earlier participants. Moreover, these participants were presented with a list ordered by the number of downloads. Additionally, to investigate the impact of social influence, participants in the second group were distributed in eight “worlds” evolving completely independently. In particular, participants in one world had no visibility about the downloads and the rankings in the other worlds.

The MusicLab provides an experimental testbed of measuring the unpredictability of cultural markets. By observing the evolution of different words given the same initial conditions, the MusicLab provides unique insights on the impact of social influence and the resulting unpredictability. In particular, Salganik et al suggested that social influence contributes to both unpredictability and inequality of success, with follow-up experiments confirming these initial findings [9–12] (see also [13] for a perspective on the MusicLab paper). These experimental results received further theoretical support by the development of a generative model that reproduces the experimental data [14]. The MusicLab model, which is presented below, teased out the role of the quality and appeal of the songs and position bias in the unpredictability and inequality of the experimental market.

Together these results painted a bleak scenario for cultural markets. From the standpoint of the market, e.g., producers and consumers of cultural products, this is an inefficient situation [15–17]: Fewer cultural products are consumed overall and some high-quality products may go unnoticed, while lower-quality ones are oversold. It is thus an important open question to determine whether this situation can be remedied and how. In [6], Watts advocates the use of “measure and react” strategies to address the difficulty in making correct predictions: “Rather than predicting how people will behave and attempting to design ways to make customers behave in a particular way […] we can instead measure directly how they respond to a whole range of possibilities and react accordingly” [6]. The MusicLab provides a unique setting to validate the benefits of a “measure and react” strategy.

This paper presents a “measure and optimize” (M&O) algorithm, that is a “measure and react” algorithm which maximizes expected downloads at each decision point. In the MusicLab, the only degree of freedom is the playlist presented to an incoming participant. Each ranking policy for the playlist produces a “measure and react” algorithm. The original MusicLab experiments presented one such policy: To rank the songs by number of downloads. Our “measure and optimize” algorithm in contrast uses a performance ranking that maximizes the expected downloads globally for the incoming participant, exploiting the quality and appeal of each song, the position visibilities in the playlist, and social influence. The M&O algorithm is based on three technical insights.

1. It uses the generative MusicLab model that describes the probability of sampling a song given its quality, appeal, position in the playlist, and the downloads of all songs at some point in time.
2. It solves the resulting global optimization problem in strongly polynomial time by a reduction, which means that the algorithm scales to large instances and avoid exploring the *n*! possible rankings.
3. Since the quality of the songs is not known a priori, the algorithm learns them over time, using an often overlooked insight by Salganik et al. [8]: The fact that the popularity of a song in the independent condition is a natural measure of its quality, capturing both its intrinsic “value” and the preferences of the participants.

The M&O algorithm was evaluated on a simulated version of the MusicLab based on the model proposed in [14]; It was compared to the download ranking policy used in the original MusicLab experiments [8] and a random ranking policy. These ranking policies were evaluated both under the social influence and independent conditions.

Regarding market efficiency, the computational results contain two important highlights:

1. The M&O algorithm produces a significant increase in expected downloads compared to other tested policies.
2. The M&O algorithm leverages both position bias and social influence to maximize downloads, providing substantial gains compared to ranking policies under the independent condition.

These performance results are robust, not an artifact of distributions and parameters used in experimental setting. Indeed, our theoretical results show that a performance-ranking policy always benefits from social influence and outperforms all policies not using social influence in expectation regardless of the distributions for the appeal and quality of the songs. The performance results also shed light on recent results that demonstrated the benefits of position bias in the absence of social influence [18]. Our computational results show that a performance-ranking policy leverages both social influence and position visibility and that the improvements in expected downloads are cumulative.

Regarding unpredictability, our results are also illuminating. They highlight that the performance-ranking policy often identifies “blockbusters”, which are the primary sources of the increased market efficiency. This provides a sharp contrast to the download ranking, which cannot identify these blockbusters reliably. Moreover, even in a worst-case scenario where the appeal is negatively with quality, our computational results show that the performance ranking brings significant benefits over the download ranking.

Finally, it is important to emphasize that the performance-ranking policy is general-purpose and can be applied to a variety of cultural markets, including books and movies, where product appeal, position visibility, and social influence can be optimized jointly.

## Results

We first describe the MusicLab model proposed in [14], the experimental setting, and a presentation of the various ranking policies. We then show how to estimate the qualities of the songs. We then report the core computational results about the market efficiency, unpredictability, and inequality produced by the various ranking policies. We finally prove that social influence always helps the performance ranking in maximizing the expected downloads. As a result, the performance ranking under social influence outperforms all ranking policies under the independent condition.

### The MusicLab Model

The descriptive model for the MusicLab [14] is based on the data collected during the actual experiments and is accurate enough to reproduce the conclusions in [8] through simulation. The model is defined in terms of a market composed of *n* songs. Each song *i* ∈ {1, …, *n*} is characterized by two values:

1. Its *appeal* *A*_*i*_ which represents the inherent preference of listening to song *i* based only on its name and its band;
2. Its *quality* *q*_*i*_ which represents the conditional probability of downloading song *i* given that it was sampled.

The MusicLab experiments present each participant with a playlist *π*, i.e., a permutation of {1, …, *n*}. Each position *p* in the playlist is characterized by its *visibility* *v*_*p*_ which is the inherent probability of sampling a song in position *p*. Since the playlist *π* is a bijection from the positions to the songs, its inverse is well-defined and is called a ranking. Rankings are denoted by the letter *σ* in the following, *π*_*i*_ denotes the song in position *i* of the playlist *π*, and *σ*_*i*_ denotes the position of song *i* in the ranking *σ*. Hence *v*_*σ*_*i*__ denotes the visibility of the position of song *i*. The model specifies the probability of listening to song *i* at time *k* given a playlist *σ* as

$$\begin{array}{c} p_{i,k}\left(\sigma \right)=\frac{v_{\sigma_{i}}\left(\alpha A_{i}+D_{i,k}\right)}{\sum_{j=1}^{n}v_{\sigma_{j}}\left(\alpha A_{j}+D_{j,k}\right)}, \end{array}$$

where *D*_*i*,*k*_ is the number of downloads of song *i* at time *k* and *α* > 0 is a scaling factor which is the same for all songs. Observe that the probability of sampling a song depends on its position in the playlist, its appeal, and the number of downloads at time *k*. As an experiment proceeds, the number of downloads dominates the appeal of the song at a rate dictated by *α*.

When simulating the independent condition, the download counts *D*_*i*,*k*_ in the probability *p*_*i*,*k*_(*σ*) becomes zero and hence the probability of listening to song *i* only depends on the appeals and visibilities of the songs. We obtain a probability

$$\begin{array}{c} p_{i}\left(\sigma \right)=\frac{v_{\sigma_{i}}A_{i}}{\sum_{j=1}^{n}v_{\sigma_{j}}A_{j}} \end{array}$$

### The Experimental Setting

The experimental setting in this paper uses an agent-based simulation to emulate the MusicLab. Each simulation consists of *N* iterations and, at each iteration *k*,

1. the simulator randomly selects a song *i* according to the probability distribution specified by the *p*_*j*.*k*_’s.
2. the simulator randomly determines, with probability *q*_*i*_, whether selected song *i* is downloaded; If the song is downloaded, then the simulator increases the download counts of song *i*, i.e., *D*_*i*,*k*+1_ = *D*_*i*,*k*_+1.

The “measure and react” algorithms studied in this paper monitors the above process (e.g., to estimate song quality). Every *r* iterations, the algorithms compute a new playlist *σ* using one of the ranking policies described below. For instance, in the social influence condition of the original MusicLab experiments, the policy ranks the songs in decreasing order of download counts. The parameter *r* ≥ 1 is called the refresh rate.

**Fig 1:**
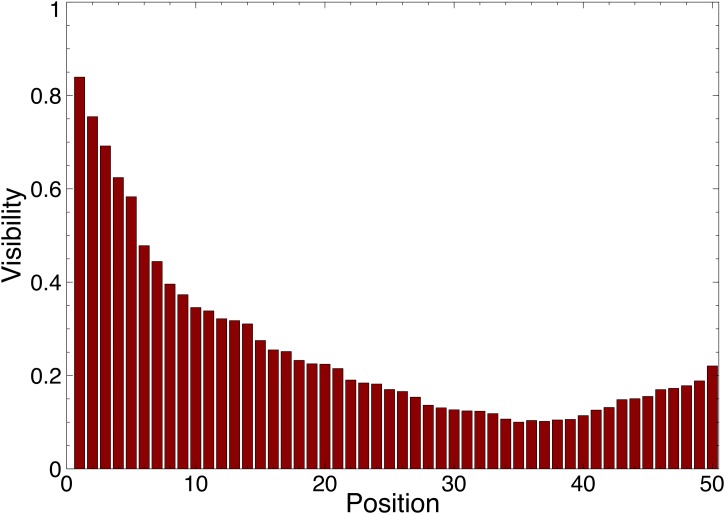
The visibility *V*_*p*_ (y-axis) of position *p* in the playlist (x-axis) where *p* = 1 is the top position and *p* = 50 is the bottom position of the playlist which is displayed in a single column.

The experimental setting tries to be close to the MusicLab experiments and considers 50 songs and simulations with 20,000 steps. The songs are displayed in a single column. The analysis in [14] indicated that participants are more likely to sample songs higher in the playlist. More precisely, the visibility decreases with the playlist position, except for a slight increase at the bottom positions. Fig. 1 depicts the visibility profile based on these guidelines, which is used in all computational experiments in this paper.

**Fig 2:**
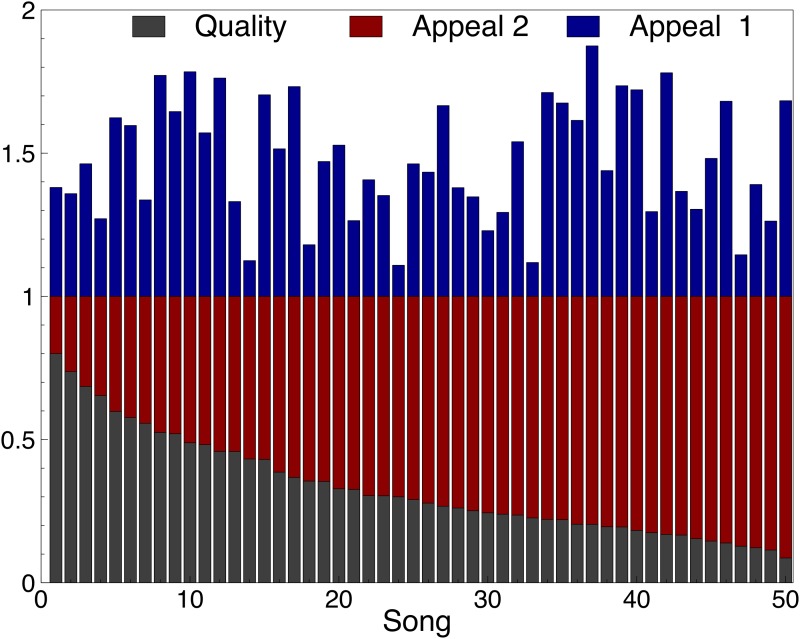
The Quality *q*_*i*_ (gray) and Appeal *A*_*i*_ (red and blue) of song *i* in the two settings. The settings only differ in the appeal of songs, and not in the quality of songs. In the first setting, the quality and the appeal for the songs were chosen independently according to a Gaussian distribution normalized to fit between 0 and 1. The second setting explores an extreme case where the appeal is negatively correlated with the quality used in setting 1. In this second setting, the appeal and quality of each song sum to 1.

The paper also uses two settings for the quality and appeal of each song, which are depicted in Fig. 2:

1. In the first setting, the quality and the appeal for the songs were chosen independently according to a Gaussian distribution normalized to fit between 0 and 1.
2. The second setting explores an extreme case where the appeal of a song is negatively correlated with its quality. The quality of each song is the same as in the first setting but the appeal is chosen such that the sum of appeal and quality is exactly 1.

The experimental results were obtained by running *W* = 400 simulations.

### The Ranking Policies

This section reviews a number of ranking policies used to influence the participants. Each policy updates the order of the songs in the playlist and thus defines a different “measure and react” algorithm that uses the policy to present a novel playlist after each refresh.

The first policy is called the *download ranking* [8]: At iteration *k*, it simply orders the songs by the number of downloads *D*_*i*,*k*_ and assigns the positions 1..*n* in that order to obtain the $\sigma_{k}^{d}$. Note that downloads do not necessarily reflect the inherent quality of the songs, since they depend on how many times the songs were listened to.

The second policy is the *performance ranking* which maximizes the expected number of downloads at each iteration, exploiting all the available information globally, i.e., the appeal, the visibility, the downloads, and the quality of the songs.

**Definition 1**. The performance ranking $\sigma_{k}^{*}$ at iteration k is defined as

$$\begin{array}{c} \sigma_{k}^{*}=\underset{\sigma \in S_{n}}{arg-max}\sum_{i=1}^{n}p_{i,k}\left(\sigma \right)\cdot q_{i}. \end{array}$$

The performance ranking uses the probability *p*_*i*,*k*_(*σ*) of sampling song *i* at iteration *k* given ranking *σ*, as well as the quality *q*_*i*_ of song *i*. Obviously, in practical situations, the song qualities are unknown. However, as mentioned in the introduction, Salganik et al. [8] argue that the popularity of a song (at least in the independent condition) can be used as a measure of its quality. Hence, the quality *q*_*i*_ in the above expression can be replaced by its approximation ${\hat{q}}_{i,k}$ at iteration *k* as discussed later in the paper.

It is not obvious a priori how to compute the performance ranking: In the worst case, there are *n*! rankings to explore. Fortunately, the performance ranking can be computed efficiently (in strongly polynomial time) through a reduction to the *Linear Fractional Assignment Problem*.

**Theorem 1**. *The performance ranking can be computed in strongly polynomial time*.

*Proof*. We show that the performance ranking problem can be polynomially reduced to the Linear Fractional Assignment Problem (LFAP). The LFAP, which we state below, is known to be solvable in polynomial time in the strong sense [19, 20].

A LFAP input is a bipartite graph *G* = (*V*_1_,*V*_2_,*E*) where ∣*V*_1_∣ = ∣*V*_2_∣ = *n*, and a cost *c*_*ij*_ and a weight *d*_*ij*_ > 0 for each edge (*i*,*j*) ∈ *E*. The LFAP amounts to finding a perfect matching *M* minimizing

$$\begin{array}{c} \frac{\sum_{(i,j)\in M}c_{ij}}{\sum_{(i,j)\in M}d_{ij}}. \end{array}$$

To find the performance ranking, it suffices to solve the LFAP problem defined over a bipartite graph where *V*_1_ represents the songs, *V*_2_ represents the positions, *c*_*i*,*j*_ = −*v*_*j*_(*αA*_*i*_+*D*_*i*_)*q*_*i*_ and *d*_*ij*_ = *v*_*j*_(*αA*_*i*_+*D*_*i*_). This LFAP instance determines a perfect matching *M* such that $\frac{-\sum_{(i,j)\in M}v_{j}(\alpha A_{i}+D_{i})q_{i}}{\sum_{(i,j)\in M}v_{j}(\alpha A_{i}+D_{i})}$ is minimized. The result follows since this is a polynomial reduction.

In the following, we use D-rank and P-rank to denote the “measure and react” algorithms using the download and performance rankings respectively. We also annotate the algorithms with either si or in to denote whether they are used under the social influence or the independent condition. For instance, P-rank(SI) denotes the algorithm with the performance ranking under the social influence condition, while P-rank(IN) denotes the algorithm with the performance ranking under the independent condition. We also use rand-rank to denote the algorithm that simply presents a random playlist at each iteration. Our proposed M&O algorithm is P-rank(SI).

Under the independent condition, the optimization problem is the same at each iteration as the probability *p*_*i*_(*σ*) does not change over time. Since the performance ranking maximizes the expected downloads at each iteration, it dominates all other “measure and react” algorithms under the independent condition.

**Proposition 1** (Optimality of Performance Ranking). *Given specified songs appeal and quality*, P-rank(IN) *is the optimal “measure and react” algorithm under the independent condition*.

### Recovering the Songs Quality

This section describes how to recover songs quality in the MusicLab. It shows that “measure and react” algorithms quickly recover the real quality of the songs.

As mentioned several times already, Salganik et al. [8] stated that the popularity of a song in the independent condition is a natural measure of its quality and captures both its intrinsic “value” and the preferences of the participants. To approximate the quality of a song, it suffices to sample the participants. This can be simulated by using a Bernoulli sampling based on real quality of the songs. The predicted quality ${\hat{q}}_{i}$ of song *i* is obtained by running *m* independent Bernoulli trials with probability *q*_*i*_ of success, i.e.,

$$\begin{array}{c} {\hat{q}}_{i}=\frac{k}{m}, \end{array}$$

where *k* is the number of successes over the *m* trials. The law of large numbers states that the prediction accuracy increases with the sampling size. For a large enough sampling size, ${\hat{q}}_{i}$ has a mean of *q*_*i*_ and a variance of *q*_*i*_(1−*q*_*i*_). This variance has the desirable property that the quality of a song with a more ‘extreme’ quality (i.e., a good or a bad song) is recovered faster than those with average quality. In addition, “measure and react” algorithms merge additional information about downloads into the prediction as the experiment proceeds: At step *k*, the approximate quality of song *i* is given by

$$\begin{array}{c} {\hat{q}}_{i,k}=\frac{{\hat{q}}_{i,0}\cdot m+D_{i,k}}{m+S_{i,k}}, \end{array}$$

where *m* is the initial sample size and *S*_*i*,*k*_ the number of samplings of song *i* up to step *k*.

**Fig 3:**
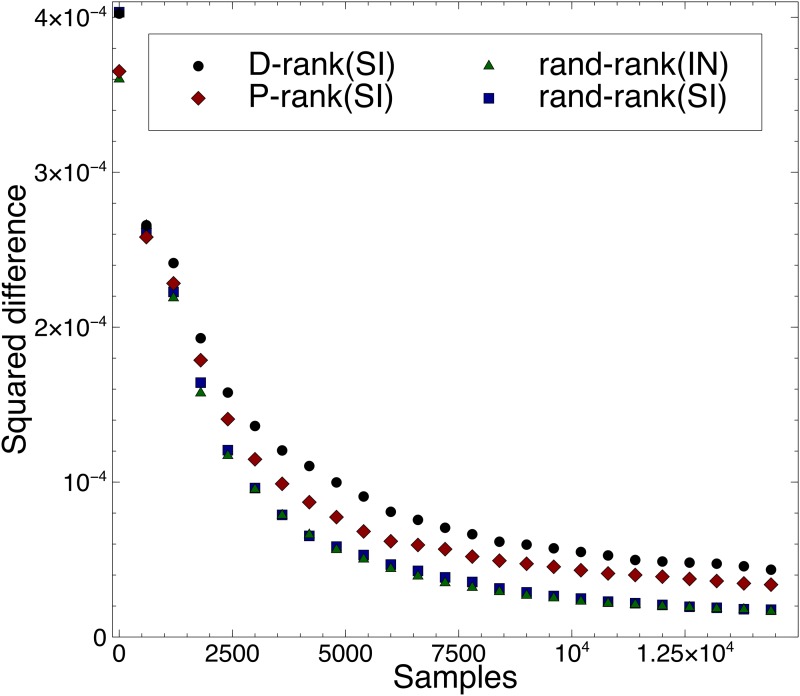
Average Squared Difference of Inferred Quality over Time for Different Rankings. The figure reports the average squared difference $\sum_{i=1}^{n}\frac{{\left({\hat{q}}_{i,k}-q_{i,k}\right)}^{2}}{n}$ between the song quality and their predictions for the various ranking policies under the social influence and the independent conditions.

Fig. 3 presents experimental results about the accuracy of the quality approximation for different rankings, assuming an initial sampling set of size 10. More precisely, the figure reports the average squared difference

$$\begin{array}{c} \sum_{i=1}^{n}\frac{{\left({\hat{q}}_{i,k}-q_{i}\right)}^{2}}{n} \end{array}$$

between the song quality and their predictions for the various ranking policies under the social influence and the independent conditions. In all cases, the results indicate that the songs quality is recovered quickly and accurately.

### Expected Market Performance

**Fig 4:**
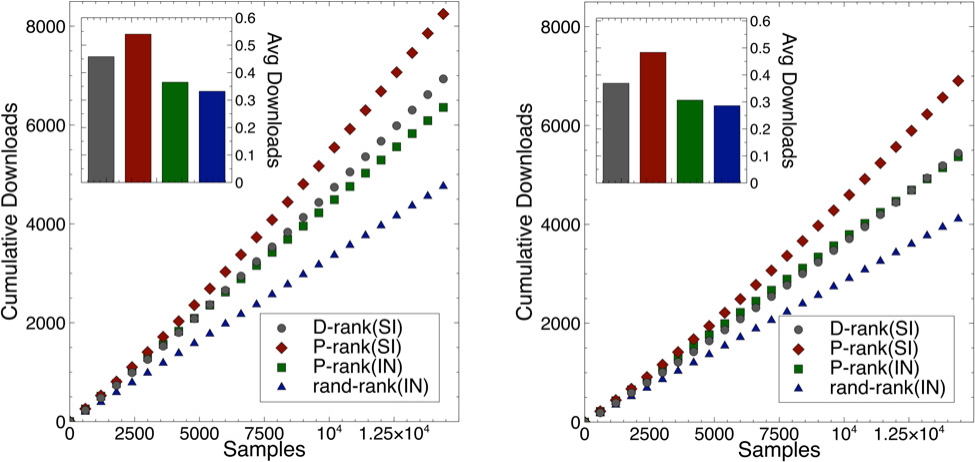
The Number of Downloads over Time for the Various Rankings. The x-axis represents the number of song samplings and the y-axis represents the average number of downloads over all experiments. The left figure depicts the results for the First Setting and the right figure for the second setting. On the upper left corner, the bar plot depicts the average number of downloads per sample for all rankings.

This section studies the expected performance of the various rankings under the social influence and the independent conditions. The performance ranking uses the quality approximation presented in the last section (instead of the “true” quality). Fig. 4 depicts the average number of downloads for the various rankings given the two settings presented in Fig. 2. The experimental results highlight three significant findings:

1. The *performance ranking* provides substantial gains in expected downloads compared to the *download ranking* and the *random ranking* policies. Social influence creates a Matthew effect [21] for the number of expected downloads. Both plots in Fig. 4 show significant differences between P-rank(SI) and D-rank(SI).
2. The benefits of social influence and position bias are complementary and cumulative. See the improvements of P-rank(IN) over rand-rank(IN) and of P-rank(SI) over P-rank(IN) in Fig. 4.
3. The gain of the performance ranking is also more significant on the worst-case scenario (Setting 2), where the song appeal is negatively correlated with the song quality. The expected number of downloads is lower in this worst-case scenario, but the shapes of the curves are in fact remarkably similar in the social influence and independent conditions.

In summary, the performance ranking produces significant improvements in expected downloads over the download ranking, which also dominate the random ranking. The benefits come both from position bias and social influence which provide cumulative improvements.

### Market Unpredictability

**Fig 5:**
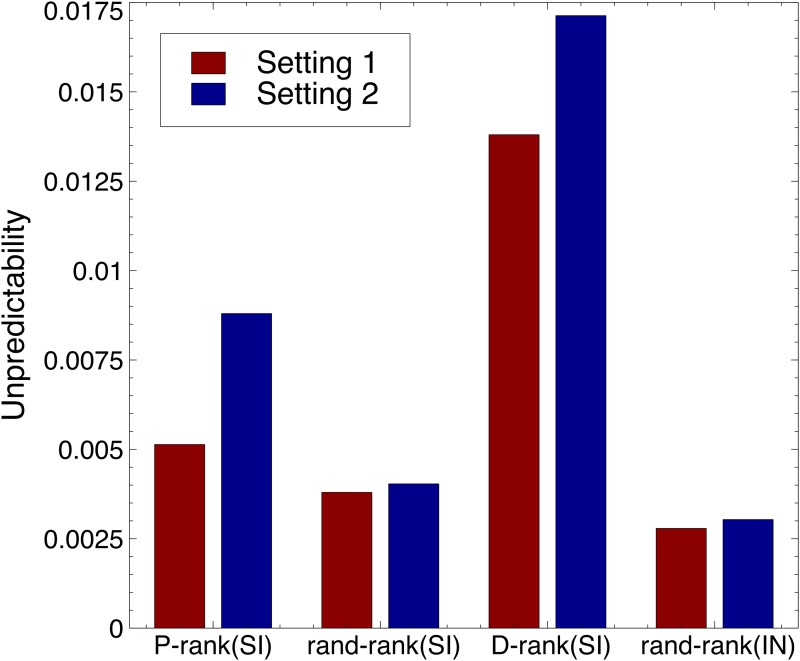
The Unpredictability of the Rankings for the Unpredictability Measure of [8]. The measure of unpredictability *u*_*i*_ for song *i* is defined as the average difference in market share *m*_*i*_ for that song between all pairs of realizations, i.e., ui=∑j=1W∑k=j+1W∣mi,j−mi,k∣/W2, where *m*_*i*,*j*_ is the market share of song *i* in world *j*. The overall unpredictability measure is the average of this measure over all *n* songs, i.e., $U=\sum_{j=1}^{n}u_{i}/n.$. The left bar depicts the results for the first setting, while the right bar depicts the results for the second setting.

Fig. 5 depicts the unpredictability results using the measure proposed in [8]. The unpredictability *u*_*i*_ of song *i* is defined as the average difference in market share for that song between all pairs of realizations, i.e.,

ui=∑w=1W∑w′=w+1W|mi,w-mi,w′|/W2,

where *m*_*i*,*w*_ is the market share of song *i* in world *w*, i.e.,

$$\begin{array}{c} m_{i,j}=D_{i,N}^{j}/\sum_{k=1}^{n}D_{i,N}^{j} \end{array}$$

where $D_{i,t}^{w}$ is the number of downloads of song *i* at step *t* in world *w*. The overall unpredictability measure is the average of this measure over all *n* songs, i.e.,

$$\begin{array}{c} U=\sum_{j=1}^{n}u_{i}/n. \end{array}$$

The figure highlights three interesting results.

1. Exploiting social influence does not necessarily create an unpredictable market as shown by the random ranking and the performance ranking in the first setting.
2. The performance ranking introduces significantly less unpredicability than the download ranking. This is particularly striking in the first setting where the performance ranking introduces only negligible unpredictability in the market.
3. An unpredictable market is not necessarily an inefficient market. In the second setting, the performance ranking exploits social influence to provide significant improvements in downloads although, as a side-effect, it creates more unpredictability. Subsequent results will explain the source of this unpredictability.

### Market Inequalities

**Fig 6:**
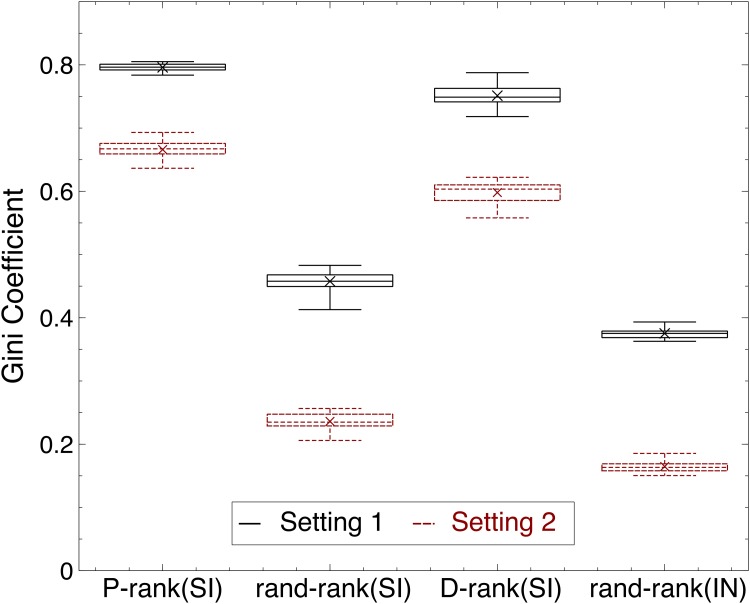
The Inequality of Success for the Rankings. The success of song *i* is defined as the market share *m*_*i*_ for that song, i.e., $m_{i,k}=D_{i,k}/\sum_{j=1}^{n}D_{j,k}$ for a given world *k*. The success inequality is defined by the Gini coefficient $G=\sum_{i=1}^{n}\sum_{j=1}^{n}|m_{i,k}-m_{j,k}|/2n\sum_{j=1}^{n}m_{j,k}$, which represents the average difference of market share for all songs. The figure depicts a boxplot with whiskers from minimum to maximum. The results for the first setting are in black, while the results for the second setting are in red and dashed line.

Fig. 6 reports the inequalities created by the various rankings in terms of the Gini coefficient. The results show that the performance and download rankings create significantly inequalities. These inequalities and the associated Matthew effects are more pronounced in the performance ranking for reasons that are explained below. Informally speaking, the inequalities created by the performance ranking are directly linked to the efficiency of the market which identifies “blockbusters”.

### Downloads Versus Quality

**Fig 7:**
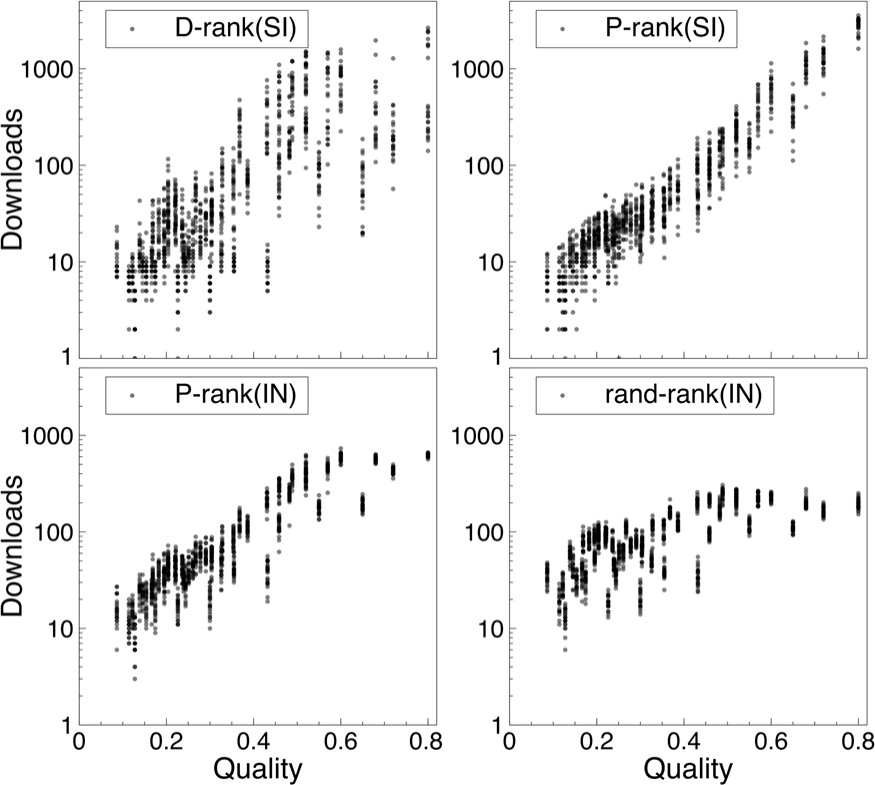
The Distribution of Downloads Versus the True Song Qualities (First Setting). The songs on the x-axis are ranked by increasing quality from left to right. Each dot is the number of downloads of a song in an experiment. Note that the y-axis is in log scale.

**Fig 8:**
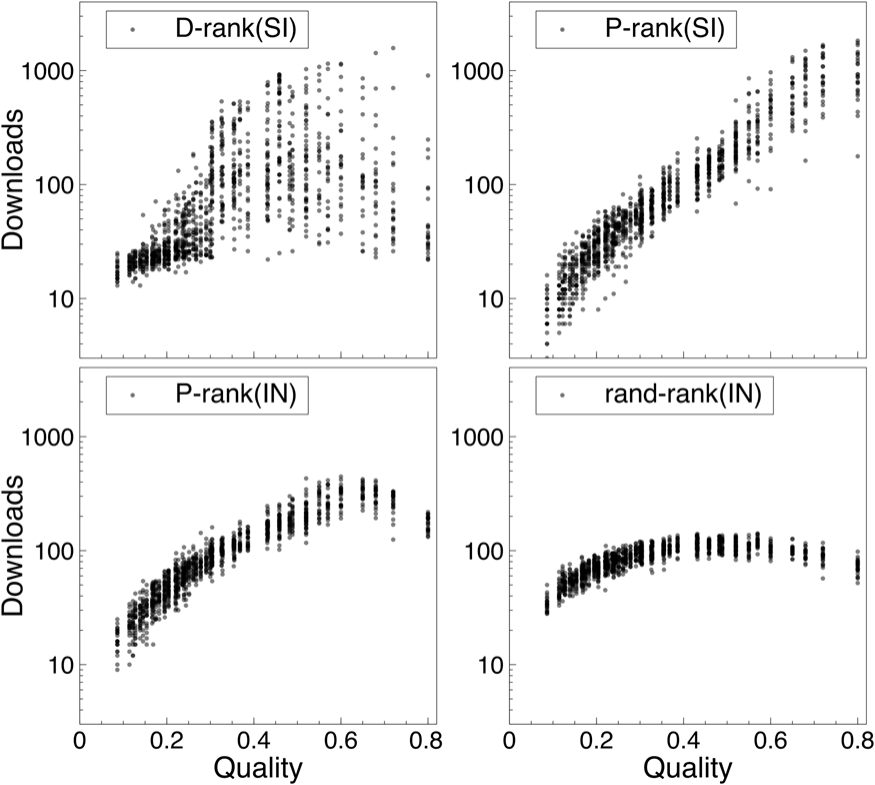
The Distribution of Downloads Versus the True Song Qualities (Second Setting). The songs on the x-axis are ranked by increasing quality from left to right. Each dot is the number of downloads of a song in an experiment. Note that the y-axis is in log scale.

Figs. 7 and 8 report the correlation between number of downloads of a song (y-axis) and its true quality (x-axis) for each of the rankings. More precisely, the figures show the distributions of the downloads of every song over the simulations. The songs are ranked in increasing quality on the x-axis and there are 400 dots for each song, representing the number of downloads in each of the experiments. As a result, the figures give a clear visual representation of what happens in the MusicLab for the various rankings, including how the downloads are distributed and the sources of unpredictability.

The results are particularly interesting. In the first setting depicted in Fig. 7, P-rank(SI) clearly isolates the best song, which has an order of magnitude more downloads than other songs (recall that the y-axis is in a log scale). When comparing with P-rank(SI) and P-rank(IN), we observe that the additional market efficiency produced by P-rank(SI) primarily comes from the downloads of this best song. Also striking is the nice monotonic download increase as a function of song quality. In contrast, the download ranking has a lot more unpredictability and has significant download counts for many other songs, even for some with considerably less quality. The variance of downloads for the best song is substantial, indicating that there are simulations in which this best song has very few downloads. This never happens with the performance ranking.

The results in Fig. 8 are for the second setting where the appeal is negatively correlated with the quality: They show that the performance ranking nicely recovers the negative appeal. Once again, the downloads increase monotonically as a function of quality, which is not the case in the download ranking or when social influence is not used (e.g., P-rank(IN)). The figure also highlights the source of unpredictability in the performance ranking. The performance ranking is not capable of isolating the best song in all worlds: Rather it isolates one of the four best songs. This contrasts with the download ranking where lower-quality songs can have substantial downloads. It is interesting to point out that the unpredictability of the performance ranking in this setting comes from songs which are not easy to distinguish: These songs have very similar values for quality and appeal.

These results shed interesting light on social influence. First, the download ranking clearly shows that social influence can transform average songs into “hits”. However, this behavior is entirely eliminated by the performance ranking in the first setting: The performance ranking clearly leverages social influence to isolate the “blockbuster”. The second setting highlights a case where even the performance ranking cannot recover the best song. This setting is of course a worst-case scenario: A terrific product with a poor appeal. However, the performance ranking still leverages social influence to promote high-quality songs: Social influence under the performance ranking is much less likely to turn an average song into a “hit” compared to the download ranking.

### The Benefits of Social Influence

We now prove that social influence always increases the number of expected downloads of the performance ranking. We show that this is true regardless of the number of songs, their appeal and quality, and the visibility values. The proof assumes that the quality of the songs has been recovered, i.e., we use *q*_*i*_ instead of ${\hat{q}}_{i,k}$. Also, for simplifying the notations, we use *a*_*i*_ = *αA*_*i*_+*D*_*i*,*t*_ and assume without loss of generality that *v*_1_ ≥ ⋯ ≥ *v*_*n*_ > 0. Proofs of the lemmas are in Appendix A.

We are interested in showing that the expected number of downloads increases over time for the performance ranking under the social influence condition. In state *t*, the probability that song *i* is downloaded for ranking *σ* is

$$\begin{array}{c} \frac{v_{\sigma_{i}}a_{i}q_{i}}{\sum v_{\sigma_{i}}a_{i}}. \end{array}$$

Hence, the expected number of downloads at time *t* for ranking *σ* is defined as

$$\begin{array}{c} 𝔼\left[D_{t}^{\sigma }\right]=\frac{\sum_{i}v_{\sigma_{i}}a_{i}q_{i}}{\sum_{i}v_{\sigma_{i}}a_{i}}. \end{array}$$

Under the social influence condition, the number of expected downloads over time can be considered as a Markov chain where state *t* + 1 only depends on state *t*. Therefore the expected number of downloads at time *t* + 1 conditional to time *t* if ranking *σ* and *σ* ^′^ are used at time *t* and *t* + 1 respectively is

$$\begin{array}{c} 𝔼\left[D_{t+1}^{\sigma ,\sigma^{\prime }}\right]=\sum_{j}\left(\frac{v_{\sigma_{j}}a_{j}q_{j}}{\sum v_{\sigma_{i}}a_{i}}\cdot \frac{\sum_{i\neq j}v_{\sigma_{i}^{\prime }}a_{i}q_{i}+v_{\sigma_{j}^{\prime }}\left(a_{j}+1\right)q_{j}}{\sum_{i\neq j}v_{\sigma_{i}^{\prime }}a_{i}+v_{\sigma_{j}^{\prime }}\left(a_{j}+1\right)}\right)+\left(1-𝔼[D_{t}^{\sigma }]\right)\cdot 𝔼\left[D_{t}^{\sigma }\right]. \end{array}$$

We first consider the case where the ranking does not change from step *t* to *t* + 1 and show that

$$\begin{array}{c} 𝔼\left[D_{t+1}^{\sigma ,\sigma }\right]\geq 𝔼\left[D_{t}^{\sigma }\right]. \end{array}$$

The following technical lemma specifies that the condition

$$\begin{array}{c} \sum_{i}\left[v_{\sigma_{i}}^{2}a_{j}\left(q_{j}-𝔼\left[D_{t}^{\sigma }\right]\right)\right]\geq 0 \end{array}$$ (1)

is sufficient for the above inequality to hold.

**Lemma 1**. *If Condition (1) holds, then* $𝔼[D_{t+1}^{\sigma }]\geq 𝔼[D_{t}^{\sigma }].$

Consider the performance ranking now. At step *t*, the performance ranking finds a permutation σ* ∈ *S*_*n*_ such that

$$\begin{array}{c} 𝔼\left[D_{t}^{\sigma^{*}}\right]=\frac{\sum_{i}v_{\sigma_{i}^{*}}a_{i}q_{i}}{\sum_{i}v_{\sigma_{i}^{*}}a_{i}}=\max_{\sigma }\frac{\sum_{i}v_{\sigma_{i}}a_{i}q_{i}}{\sum_{i}v_{\sigma_{i}}a_{i}}. \end{array}$$ (2)

By optimality of the performance ranking at each step, we have that

$$\begin{array}{c} 𝔼\left[D_{t+1}^{\sigma^{*},\sigma^{**}}\right]\geq 𝔼\left[D_{t+1}^{\sigma^{*},\sigma^{*}}\right]. \end{array}$$

where σ** is the performance ranking at step *t* + 1.

**Corollary 1**. Let σ* and σ** be the performance rankings at steps t and t + 1. If Condition (1) holds for σ*, then $𝔼[D_{t+1}^{\sigma^{**}}]\geq 𝔼[D_{t}^{\sigma^{*}}]$.

It remains to show that the performance ranking σ* at step *t* satisfies Condition (1). This is easier to show by reasoning about the playlist obtained by the performance ranking. More precisely, at step *t*, the performance ranking finds a playlist π* ∈ *S*_*n*_ such that

$$\begin{array}{c} \frac{\sum_{p}v_{p}a_{\pi_{p}^{*}}q_{\pi_{p}^{*}}}{\sum_{p}v_{p}a_{\pi_{p}^{*}}}=𝔼\left[D_{t}^{\sigma^{*}}\right] \end{array}$$ (3)

By (3), π* satisfies

$$\begin{array}{c} \sum_{p}v_{p}a_{\pi_{p}^{*}}q_{\pi_{p}^{*}}-𝔼\left[D_{t}^{\sigma^{*}}\right]\;\sum_{p}v_{p}a_{\pi_{p}^{*}}=\sum_{p}v_{p}a_{\pi_{p}^{*}}\left(q_{\pi_{p}^{*}}-𝔼\left[D_{t}^{\sigma^{*}}\right]\right)=0. \end{array}$$ (4)

Consider the function

$$\begin{array}{c} f\left(\lambda \right)=\max_{\pi }\sum_{p}v_{p}a_{\pi_{p}^{*}}\left(q_{\pi_{p}^{*}}-\lambda \right). \end{array}$$

Function *f* is concave and strictly decreasing and hence its unique zero is the optimal value $𝔼[D_{t}^{\sigma^{*}}]$. Furthermore, the optimality condition for *π* in function *f* can be characterized by a “rearrangement inequality” which states that the maximum is reached if and only if

$$\begin{array}{c} a_{\pi_{1}^{*}}\left(q_{\pi_{1}^{*}}-\lambda \right)\geq \cdots \geq a_{\pi_{n}^{*}}\left(q_{\pi_{n}^{*}}-\lambda \right)\;\forall \lambda \in ℛ. \end{array}$$

**Lemma 2**. *The performance ranking at step t produces a playlist π satisfying*

$$\begin{array}{c} a_{\pi_{1}^{*}}\left(q_{\pi_{1}^{*}}-𝔼\left[D_{t}^{\sigma^{*}}\right]\right)\geq \cdots \geq a_{\pi_{n}^{*}}\left(q_{\pi_{n}^{*}}-𝔼\left[D_{t}^{\sigma^{*}}\right]\right). \end{array}$$

Together with Equation (4), this rearrangement inequality makes it possible to prove that the performance ranking at step *t* satisfies Condition (1).

**Corollary 2**. The performance ranking σ* at step t satisfies Condition (1).

We are now in position to state our key theoretical result.

**Theorem 2**. *The expected downloads are nondecreasing over time when the performance ranking is used to select the playlist*.

*Proof*. By Corollary 2, the performance ranking satisfied Condition (1). Hence, by Corollary 1, the expected downloads at step *t* + 1 is at least as large as the the expected downloads at step *t*.

**Corollary 3**. *In expectation, the P-ranking policy under social influence achieves more downloads than the any ranking policy under the independent condition*.

This corollary follows directly from the fact that the expected downloads at each step do not change under the independent condition regardless of the ranking policy in use.

## Conclusion and Discussion

This paper presented a “measure and optimize” algorithm for the MusicLab which computes, at each refresh step, an optimal ranking of the songs, given the appeal, estimated quality, current download counts, and position visibility. This performance ranking, which maximizes the expected number of downloads, can be computed in strongly polynomial time, making the M&O algorithm highly scalable. Our experimental results reveal two key findings:

1. The M&O algorithm leverages social influence to bring significant benefits in expected downloads (see Fig. 4).
2. Social influence and position bias both bring improvements in expected downloads and their effects are cumulative. (See the improvements of P-rank(IN) over rand-rank(IN) and of P-rank(SI) over P-rank(SI) in Fig. 4).

Our theoretical results formally validate the first finding and prove that the performance ranking under social influence always achieves more expected downloads than any ranking policy under the independent condition.

Our results shed an interesting and novel light about social influence and the unpredictability it creates in cultural markets. The unpredictability coming from social influence is often presented as an undesirable property of cultural markets. *However, as our experimental and theoretical results show, social influence, when used properly, is guaranteed to help in maximizing the efficiency of the market*. So, unless predictability is a goal per se, social influence should be seen as an asset for optimizing cultural markets.

Our results also show that, in reasonable settings and with a performance ranking, the unpredictability created by the use of social influence is small (See Fig. 5). Moreover, the performance ranking identifies “blockbusters” and these “blockbusters” are the primary reason for the increased efficiency of the market (See Fig. 7). There are conditions in the MusicLab where the best song is not recovered and does not have the most downloads. This happens when the best song has a “bad” appeal and is dominated by another song. Assume, for instance, that we have only two songs and *q*_1_ > *q*_2_. If *v*_1_ *A*_1_ *q*_1_ < *v*_2_ *A*_2_ *q*_2_ (*v*_1_ > *v*_2_), the added visibility is not able to overcome the bad appeal: Song 1, despite its better quality, will be dominated by song 2. Note however that these situations can be predicted, since these dominance relationships can be identified. Equally important, social influence still helps in these settings compared to policies not using it.

The unpredictability when using the performance ranking comes from songs that are indistinguishable, i.e., *A*_*i*_ *q*_*i*_ ≈ *A*_*j*_ *q*_*j*_. In this case, social influence may favor one of the songs depending on how the process unfolds. It is interesting to point out once again that these situations can be identified a priori. Together with the dominance relationships, these equivalence relationships helps us identify the sources of unpredictability, i.e., which songs may emerge in the MusicLab. Moreover, it is conceivable that we can also control this unpredictability to ensure fairness and reduce inequalities. This is a topic we are currently investigating.

Our experiments have also shown that, contrary to the download ranking, the performance ranking does not transform average songs into “hits”. In reasonable settings, it always identifies the “blockbusters” (See Fig. 7 on Setting 1) while, in a worst-case scenario where song appeal is negatively correlated with song quality, it still promotes high-quality songs (See Fig. 7 on Setting 2). There is a certain robustness in the performance ranking which is not present in the download ranking.

A key insight from this research is the need to leverage social influence carefully. The download ranking uses social influence twice: Directly through the download counts and indirectly through the ranking. The experimental results indicate that the resulting algorithm puts too much emphasis on download counts, which are not necessarily a good indication of quality due to the sampling process. In contrast, the performance ranking balances appeal, quality, and social influence.

Observe that, in the generative model of the MusicLab, the perceived quality of a song is not affected by social influence: Only the probability of listening to the song is. Our results continue to hold when the perceived quality of a song is affected by social influence [22–24], e.g., when the perceived quality of song *i* is improved by *ϵ*_*i*_ ≥ 0, when song *i* is downloaded. Similarly, the results generalize to the case where the sampling probability is given by

$$\begin{array}{c} p_{i,k}\left(\sigma \right)=\frac{v_{\sigma_{i}}\left(\alpha A_{i}+f\left(D_{i,k}\right)\right)}{\sum_{j=1}^{n}v_{\sigma_{j}}\left(\alpha A_{j}+f\left(D_{j,k}\right)\right)}, \end{array}$$

where function *f* is positive nondecreasing.

We hope that these results will revive the debate about the consequences of social influence. Our results show that social influence is beneficial for the efficiency of the market and that much of its induced unpredictability can be controlled. It appears that a key issue is to continue building our understanding of the nature of social influence and to determine how best to use it and for which purposes: Efficiency, predictability, fairness, …

## Proofs of the Results

This lemma is important for the proof of Lemma 1.

**Lemma 3**. *For any permutation π ∈ S_n_, we have*

$$\begin{array}{c} \frac{\sum_{i}u_{i}q_{\pi_{i}}}{\sum_{i}u_{i}}\leq \frac{\sum_{i}u_{i}q_{\pi_{i}}^{2}}{\sum_{i}u_{i}q_{\sigma_{i}}}, \end{array}$$

*where u_i_* ≥ 0 *and q_i_* ≥ 0 (1 ≤ *i* ≤ *n*).

*Proof*. Without loss of generality, we rename the songs so that *π*_*i*_ = *i*. Then

$$\begin{array}{ccc} 0 & \leq & \sum_{i>j}u_{i}u_{j}{\left(q_{i}-q_{j}\right)}^{2}=\sum_{i>j}u_{i}u_{j}\left(q_{i}^{2}+q_{j}^{2}-q_{i}q_{j}-q_{i}q_{j}\right) \end{array}$$

is equivalent to

$$\begin{array}{c} \sum_{i>j}u_{i}u_{j}q_{i}q_{j}+\sum_{i>j}u_{i}u_{j}q_{i}q_{j}\leq \sum_{i>j}u_{i}u_{j}q_{i}^{2}+\sum_{i>j}u_{i}u_{j}q_{j}^{2}. \end{array}$$ (5)

Renaming indexes of the second terms of both sides yields

$$\begin{array}{c} \sum_{i>j}u_{i}u_{j}q_{i}q_{j}+\sum_{i<j}u_{i}u_{j}q_{i}q_{j}\leq \sum_{i>j}u_{i}u_{j}q_{i}^{2}+\sum_{i<j}u_{i}u_{j}q_{i}^{2} \end{array}$$

By fixing *i*, the left side of the inequality can be written as

$$\begin{array}{c} \sum_{i=1}u_{i}q_{i}\sum_{\underset{j\neq i}{j=1}}u_{j}q_{j} \end{array}$$

and the right side as

$$\begin{array}{c} \sum_{j=1}u_{j}\sum_{\underset{i\neq j}{i=1}}u_{i}q_{i}^{2} \end{array}$$

Therefore, inequality (5) can be expressed as

$$\begin{array}{c} \sum_{i=1}u_{i}q_{i}\sum_{\underset{j\neq i}{j=1}}u_{j}q_{j}\leq \sum_{i=1}u_{i}\sum_{\underset{j\neq i}{j=1}}u_{j}q_{j}^{2} \end{array}$$

Adding $\sum_{j=1}u_{j}^{2}q_{j}^{2}$ to both sides yields

$$\begin{array}{c} \sum_{i=1}u_{i}q_{i}\sum_{j=1}u_{j}q_{j}\leq \sum_{i=1}u_{i}\sum_{j=1}u_{j}q_{j}^{2} \end{array}$$

and hence

$$\begin{array}{c} \frac{\sum_{i=1}u_{i}q_{i}}{\sum_{i=1}u_{i}}\leq \frac{\sum_{j=1}u_{j}q_{j}^{2}}{\sum_{j=1}u_{j}q_{j}}. \end{array}$$

Below we present the proofs of Lemma 1 and Corollary 2.

*Proof*. (of Lemma 1) Let *a* = *a*_1_, ⋯, *a*_*n*_ represent the current state at time *t*. For ease of notation, we rename the songs so that $\sigma_{i}^{*}=i$. We also drop the ranking superscript σ* from the download notation. The optimal expected number of downloads at time *t* can be written as

$$\begin{array}{c} 𝔼\left[D_{t}\right]=\frac{\sum_{i}v_{i}a_{i}q_{i}}{\sum_{i}v_{i}a_{i}}=\lambda^{*}. \end{array}$$

and the expected number of downloads in time *t* + 1 conditional to time *t* is

$$\begin{array}{c} 𝔼\left[D_{t+1}\right]=\sum_{j}\left(\frac{v_{j}a_{j}q_{j}}{\sum v_{i}a_{i}}\cdot \frac{\sum_{i\neq j}v_{i}a_{i}q_{i}+v_{j}\left(a_{j}+1\right)q_{j}}{\sum_{i\neq j}v_{i}a_{i}+v_{j}\left(a_{j}+1\right)}\right)+\left(1-\frac{\sum_{i}v_{i}a_{i}q_{i}}{\sum_{i}v_{i}a_{i}}\right)\cdot \frac{\sum_{i}v_{i}a_{i}q_{i}}{\sum_{i}v_{i}a_{i}}\\ =\sum_{j}\left(\frac{v_{j}a_{j}q_{j}}{\sum v_{i}a_{i}}\cdot \frac{\sum_{i}v_{i}a_{i}q_{i}+v_{j}q_{j}}{\sum_{i}v_{i}a_{i}+v_{j}}\right)+\left(1-\frac{\sum_{j}v_{j}a_{j}q_{j}}{\sum_{i}v_{i}a_{i}}\right)\cdot \lambda^{*}. \end{array}$$

Proving

$$\begin{array}{c} 𝔼\left[D_{t+1}\right]\geq 𝔼\left[D_{t}\right] \end{array}$$ (6)

amounts to showing that

$$\begin{array}{c} \sum_{j}\left(\frac{v_{j}a_{j}q_{j}}{\sum v_{i}a_{i}}\cdot \frac{\sum_{i}v_{i}a_{i}q_{i}+v_{j}q_{j}}{\sum_{i}v_{i}a_{i}+v_{j}}\right)+\left(1-\frac{\sum_{j}v_{j}a_{j}q_{j}}{\sum_{i}v_{i}a_{i}}\right)\cdot \lambda^{*}\geq \lambda^{*}. \end{array}$$

which reduces to proving

$$\begin{array}{c} \frac{1}{\sum_{i}v_{i}a_{i}}\sum_{j}\left[\frac{v_{j}^{2}a_{j}q_{j}}{\sum_{i}v_{i}a_{i}+v_{j}}\left(q_{j}-\lambda^{*}\right)\right]\geq 0 \end{array}$$

or, equivalently,

$$\begin{array}{c} \sum_{j}\left[\frac{v_{j}^{2}a_{j}q_{j}}{\sum_{i}v_{i}a_{i}+v_{j}}\left(q_{j}-\lambda^{*}\right)\right]\geq 0. \end{array}$$

Now there exists a positive constant *c* > 0 such that

$$\begin{array}{c} \frac{\sum_{j}\left[v_{j}^{2}a_{j}q_{j}\left(q_{j}-\lambda^{*}\right)\right]}{\sum_{i}v_{i}a_{i}+c}\geq 0\Rightarrow \sum_{j}\left[\frac{v_{j}^{2}a_{j}q_{j}}{\sum_{i}v_{i}a_{i}+v_{j}}\left(q_{j}-\lambda^{*}\right)\right]\geq 0 \end{array}$$

which is equivalent to

$$\begin{array}{c} \sum_{j}\left[v_{j}^{2}a_{j}q_{j}\left(q_{j}-\lambda^{*}\right)\right]\geq 0\Rightarrow \sum_{j}\left[\frac{v_{j}^{2}a_{j}q_{j}}{\sum_{i}v_{i}a_{i}+v_{j}}\left(q_{j}-\lambda^{*}\right)\right]\geq 0 \end{array}$$

and allows us to avoid considering the *v*_*j*_’s in the denominator. By Lemma 3, using the transformation $u_{i}=v_{i}^{2}a_{i}$, we obtain

$$\begin{array}{c} \frac{\sum_{i}v_{i}^{2}a_{i}q_{i}^{2}}{\sum_{i}v_{i}^{2}a_{i}q_{i_{i}}}\geq \frac{\sum_{i}v_{i}^{2}a_{i}q_{i}}{\sum_{i}v_{i}^{2}a_{i}}\geq \lambda^{*} \end{array}$$

and

$$\begin{array}{c} \sum_{j}\left[v_{j}^{2}a_{j}\left(q_{j}-\lambda^{*}\right)\right]\geq 0\Rightarrow \sum_{j}\left[v_{j}^{2}a_{j}q_{j}\left(q_{j}-\lambda^{*}\right)\right]\geq 0. \end{array}$$

*Proof of Corollary 2*. At step *t*, the performance ranking finds a playlist π* ∈ *S*_*n*_ such that

$$\begin{array}{c} \frac{\sum_{p}v_{p}a_{\pi_{p}^{*}}q_{\pi_{p}^{*}}}{\sum_{p}v_{p}a_{\pi_{p}^{*}}}=𝔼\left[D_{t}^{\sigma^{*}}\right]. \end{array}$$ (7)

By (3), π* satisfies

$$\begin{array}{c} \sum_{p}v_{p}a_{\pi_{p}^{*}}q_{\pi_{p}^{*}}-𝔼\left[D_{t}^{\sigma^{*}}\right]\;\sum_{p}v_{p}a_{\pi_{p}^{*}}=\sum_{p}v_{p}a_{\pi_{p}^{*}}\left(q_{\pi_{p}^{*}}-𝔼\left[D_{t}^{\sigma^{*}}\right]\right)=0. \end{array}$$ (8)

Let π* the playlist that corresponds to the ranking σ* and let

$$\begin{array}{c} x_{i}=v_{i}a_{\pi_{i}^{*}}\left(q_{\pi_{i}^{*}}-𝔼\left[D_{t}^{\sigma^{*}}\right]\right). \end{array}$$

Since *v*_1_ ≥ ⋯ ≥ *v*_*n*_ > 0, we have that *x*_1_ ≥ ⋯ ≥ *x*_*n*_ and, by (8),

$$\begin{array}{c} \sum_{i}x_{i}=0. \end{array}$$

Split the *x*_*i*_’s into positive (1, …, *p*) and negative (*p* + 1, …, *n*) numbers. Then

$$\begin{array}{c} \sum_{i=1}^{p}x_{i}=\sum_{i=p+1}^{n}\left(-x_{i}\right) \end{array}$$

and, since *v*_*p*_ ≥ *v*_*p*+1_,

$$\begin{array}{c} \sum_{i=1}^{p}v_{p}x_{i}\geq \sum_{i=p+1}^{n}v_{p+1}\left(-x_{i}\right) \end{array}$$

Now, since the *v*_*i*_ are decreasing,

$$\begin{array}{c} \sum_{i=1}^{p}v_{i}x_{i}\geq \sum_{i=1}^{p}v_{p}x_{i} \end{array}$$

and

$$\begin{array}{c} \sum_{i=p+1}^{n}v_{p+1}\left(-x_{i}\right)\geq \sum_{i=p+1}^{n}v_{i}\left(-x_{i}\right) \end{array}$$

and the results follows.

## References

[1] Bielby WT, Bielby DD. All hits are flukes: Institutionalized decision making and the rhetoric of network prime-time program development. American Journal of Sociology. 1994;99(5):1287–1313.

[2] Caves RE. Creative industries: Contracts between art and commerce. 20. Harvard University Press; 2000.

[3] De Vany A. Hollywood Economics: How Extreme Uncertainty Shames the Film Industry. Routledge; 2011.

[4] Hirsch PM. Processing fads and fashions: An organization-set analysis of cultural industry systems. American journal of sociology. 1972:639–659.

[5] Peterson RA, Berger DG. Entrepreneurship in Organizations: Evidence from the Popular Music Industry. Administrative Science Quarterly. 1971;16(1). 10.2307/2391293

[6] Watts DJ. Everything Is Obvious: How Common Sense Fails Us. Random House LLC; 2012.

[7] Eliashberg J, Shugan SM. Film critics: Influencers or predictors? Journal of Marketing. 1997;61(2). 10.2307/1251831

[8] Salganik MJ, Dodds PS, Watts DJ. Experimental study of inequality and unpredictability in an artificial cultural market. Science. 2006;311(5762):854–856. 16469928 10.1126/science.1121066

[9] Salganik MJ, Watts DJ. Web-Based Experiments for the Study of Collective Social Dynamics in Cultural Markets. Topics in Cognitive Science. 2009;1(3):439–468. 10.1111/j.1756-8765.2009.01030.x 25164996 PMC4151995

[10] Salganik MJ, Watts DJ. Leading the herd astray: An experimental study of self-fulfilling prophecies in an artificial cultural market. Social Psychology Quarterly. 2008;71(4):338–355. 10.1177/019027250807100404 PMC3785310 24078078

[11] Muchnik L, Aral S, Taylor SJ. Social influence bias: A randomized experiment. Science. 2013;341(6146):647–651. 10.1126/science.1240466 23929980

[12] van de Rijt A, Kang SM, Restivo M, Patil A. Field experiments of success-breeds-success dynamics. Proceedings of the National Academy of Sciences. 2014;111(19):6934–6939. 10.1073/pnas.1316836111 PMC4024896 24778230

[13] Hedström P. Experimental macro sociology: Predicting the next best seller. Science. 2006;311(5762):786–787. 10.1126/science.1124707 16469907

[14] Krumme C, Cebrian M, Pickard G, Pentland S. Quantifying social influence in an online cultural market. PloS one. 2012;7(5):e33785 10.1371/journal.pone.0033785 22590493 PMC3348939

[15] Shiller RJ. Market volatility. MIT press; 1992.

[16] Shleifer A. Inefficient markets: An introduction to behavioral finance. Oxford university press; 2000.

[17] Jensen MC. Some anomalous evidence regarding market efficiency. Journal of financial economics. 1978;6(2):95–101. 10.1016/0304-405X(78)90025-9

[18] Lerman K, Hogg T. Leveraging Position Bias to Improve Peer Recommendation. PLOS ONE. 2014;9(6):1–8. 10.1371/journal.pone.0098914 PMC4053364 24919071

[19] Shigeno M, Saruwatari Y, Matsui T. An algorithm for fractional assignment problems. Discrete Applied Mathematics. 1995;56(2):333–343. 10.1016/0166-218X(93)00094-G

[20] Kabadi SN, Punnen AP. A strongly polynomial simplex method for the linear fractional assignment problem. Operations Research Letters. 2008;36(4):402–407. 10.1016/j.orl.2007.12.001

[21] Rigney D. The Matthew Effect. Columbia University Press; 2010.

[22] Lorenz J, Rauhut H, Schweitzer F, Helbing D. How social influence can undermine the wisdom of crowd effect. Proceedings of the National Academy of Sciences. 2011;108(22):9020–9025. 10.1073/pnas.1008636108 PMC3107299 21576485

[23] Farrell S. Social influence benefits the wisdom of individuals in the crowd. Proceedings of the National Academy of Sciences. 2011;108(36):E625–E625. 10.1073/pnas.1109947108 PMC3169111 21876181

[24] Rauhut H, Lorenz J, Schweitzer F, Helbing D. Reply to Farrell: Improved individual estimation success can imply collective tunnel vision. Proceedings of the National Academy of Sciences. 2011;108(36):E626–E626. 10.1073/pnas.1111007108

